# A single center retrospective study of vNOTES hysterectomy, laparoscopic hysterectomy and vaginal hysterectomy operations

**DOI:** 10.1097/MD.0000000000040881

**Published:** 2024-12-13

**Authors:** Fatmanur Mollahüseyinoğlu Küllaç, Ayşe Zehra Özdemir, Çağlanur Yildiz

**Affiliations:** a Bafra State Hospital, Obstetrics-Gynecology Department, Samsun, Turkey; b Ondokuz Mayis University Faculty of Medicine, Obstetrics-Gynecology Department, Samsun, Turkey.

**Keywords:** hysterectomy, laparoscopic hysterectomy, transvaginal natural orifice transluminal endoscopic surgery (vNOTES), vaginal hysterectomy

## Abstract

In our study, we aimed to retrospectively compare vNOTES hysterectomy, a new method, with a vaginal hysterectomy (VH) and total laparoscopic hysterectomy (TLH). Our study included 186 hysterectomy cases (62 vNOTES hysterectomy, 62 VH, and 62 TLH) with benign indications diagnosed between 2021 and 2022. Our study is a retrospective and single-center study. Three surgical techniques were evaluated comparatively according to demographic characteristics and surgical results. The least hemoglobin (Hb) decline and the longest mean operation time were observed in the vNOTES hysterectomy group. When discharge times were compared, it was observed that the vNOTES hysterectomy group was discharged significantly shorter than the TLH group (*P*: .022 < .05). The lowest 6th-hour pain score of 1.53 was observed in the vNOTES hysterectomy group. History of pelvic surgery and cesarean section were higher in the vNOTES hysterectomy group than in the VH group. Also, oophorectomy and salpingectomy rates were higher in the vNOTES hysterectomy group than in the VH group. When intraoperative complications were evaluated, bladder perforation was encountered in one vNOTES hysterectomy and two VH operations, and bleeding was observed in one VH operation. The VH group was the least costly operation compared to vNOTES hysterectomy and TLH (*P*: .003 < .05, *P*: .01 < .05). Aesthetic results can be achieved for patients by using the vaginal route. With all these results, we believe that vNOTES hysterectomy is a safe alternative to TLH and VH.

## 1. Introduction

Hysterectomy is the most common non-obstetric operation performed in obstetrics and gynecology clinics. Benign causes such as abnormal uterine bleeding, uterine descensus, endometriosis, and myoma uteri constitute the majority of indications for hysterectomy.^[1]^ After the hysterectomy decision is taken, the choice of surgery may vary depending on the patient’s clinic, the surgeon’s experience, anatomical suitability, and the adequacy of the equipment of the health center where the operation will be performed. Hysterectomy methods are divided into two as laparotomy and minimally invasive surgery by Aarts et al in the Cochrane Database.^[2]^ Minimally invasive surgery is defined as vaginal route, laparoscopy, robot-assisted laparoscopy, and laparoscopy-assisted vaginal hysterectomy.

Today, total laparoscopic hysterectomy (TLH) are more frequently performed than vaginal hysterectomy (VH) because they provide easier access to the adnexa and increase the surgeon’s field of view.^[2]^ Despite this, VH is still the most cost-effective, fastest healing, and has the best cosmetic results. vNOTES (vaginal assisted natural orifice transluminal endoscopic surgery) hysterectomy, a new technique developed for ease of access to the adnexa and good cosmetic results, is now frequently preferred by gynecologists. The first vNOTES hysterectomy operation was performed by Su et al with a series of 16 cases.^[3]^ In the following years, there have been many studies comparing vNOTES hysterectomy with TLH and VH separately.^[4–6]^ Its application in gynecologic oncology is still relatively new, nevertheless. Few cases or case reports are included in studies on vNOTES in gynecologic oncology.^[7–9]^

The purpose of this study was to retrospectively compare vNOTES hysterectomy operations carried out in our center with VH and TLH cases.

## 2. Materials and methods

This is a single-center study including a retrospective review of all 62 vNOTES hysterectomies performed at Ondokuz Mayis University Faculty of Medicine, and 62 VHs and 62 TLHs with benign indications, for a total of 186 hysterectomy cases between January 1, 2021, and December 1, 2022. The last 62 operations performed retrospectively were included in the study to avoid bias in patient selection in the VH and TLH groups. Study data were obtained from hospital records. Pain scores were obtained by asking the patients individually. Demographic characteristics included: age, body mass index (BMI), parity, history of cesarean section, history of pelvic surgery, indications for hysterectomy. We also included surgical results: blood loss (the difference between preoperative and postoperative Hb, operating time (minutes), uterine weight (grams), operating cost (Turkish Lira), complications, additional surgical procedure, conversion to laparoscopy or laparotomy and postoperative 6th hour pain score. Whether or not to perform andexial surgery was decided according to the patient’s age, oncologic risks, and the presence or absence of an adnexial mass.

Patients operated for malignancy and endometriosis were not included in the study. The pathology results of the patients included in the study were benign. The operations in our study were performed by three different surgeons (A.Z., M.Ö., and M.T.) and the surgical technique for hysterectomy was left to the surgeon’s preference. The average annual number of hysterectomies performed by each of the three surgeons is over one hundred.

The indications of the patients included in the study were abnormal uterine bleeding, myoma uteri, ovarian cyst, pelvic organ prolapse, and cervical pathologies. vNOTES hysterectomy group consisted of patients with no descensus uteri or grade 1 to 2 descensus. A history of cesarean section and/or pelvic surgery was not considered a contraindication for all three surgical techniques. All patients in the vNOTES hysterectomy group included in our study were multiparous. Two patients in the TLH group and 2 patients in the VH group were nulliparous. Before the operation, a detailed anamnesis was taken from the patients, and abdominal and pelvic examination was performed and evaluated with necessary imaging (ultrasonography and/or magnetic resonance imaging, tomography).

Among these patients, 71 patients were operated for abnormal uterine bleeding, 31 patients for myoma uteri, 18 patients for ovarian cysts, 57 patients for pelvic organ prolapse, and 9 patients for cervical pathology.

The study was performed in accordance with the Declaration of Helsinki and was approved by the local ethics committee of Ondokuz Mayis University (Protocol Number KAEK 2022/400, approved in September 2022). A written informed consent was obtained from all subjects enrolled in the present study.

### 2.1. Surgical procedure

#### 2.1.1. Laparoscopic hysterectomy

Patients undergoing laparoscopic hysterectomy were prepared in the lithotomy position under general anesthesia. Patients were given 2 g of cefazolin sodium prophylactically before the operation. The operation field was sterilized with povidone-iodine solution and covered sterilely.

A uterine manipulator was implanted into the uterus to ensure uterus retraction before the start of the operation. Clermont Ferrand type uterus manipulator was employed in our laparoscopic hysterectomy cases included in the study.

The abdomen was entered with a 10 mm trocar below the umbilicus and the pneumoperitoneum was created with 4 L of CO_2_. Afterward, the patient was placed in the Trendelenburg position and the abdomen was entered with a 10mm trocar above the symphysis and the right and left paramedian with 5 mm trocars.

Hysterectomy was performed using bipolar and monopolar energy. The uterus was resected vaginally in every patient in the study.

Vaginal cuff repair was sutured continuously with a no-1 absorbable self-locking suture or individually with a no-1 absorbable synthetic monofilament suture.

#### 2.1.2. Vaginal hysterectomy

Patients undergoing vaginal hysterectomy were prepared in the high lithotomy position under general anesthesia. Before the operation, 2 g of cefazolin sodium was administered intravenously as prophylactic. The operation field was sterilized with a povidone-iodine solution and covered sterilely.

After the cervix was grasped at the 12 and 6 lines with 2 single-toothed tenaculum, a paracervical incision was made with the help of cautery or scalpel. The bladder in front and rectum in the back were detached and the visceral peritoneum was entered. Hysterectomy was performed with the help of bipolar energy or by cutting and suturing the ligaments.

The vaginal cuff was sutured individually or continuously with a no-1 absorbable synthetic monofilament suture.

#### 2.1.3. vNOTES hysterectomy

vNOTES patients who were to be subjected to hysterectomy were prepared in the high lithotomy position under general anesthesia. Before the operation, 2 g of cefazolin sodium was administered intravenously as prophylactic. The operation field was sterilized with a povidone-iodine solution and covered sterilely.

The initial steps of the operation were the same as in a vaginal hysterectomy.

**Figure 1.**
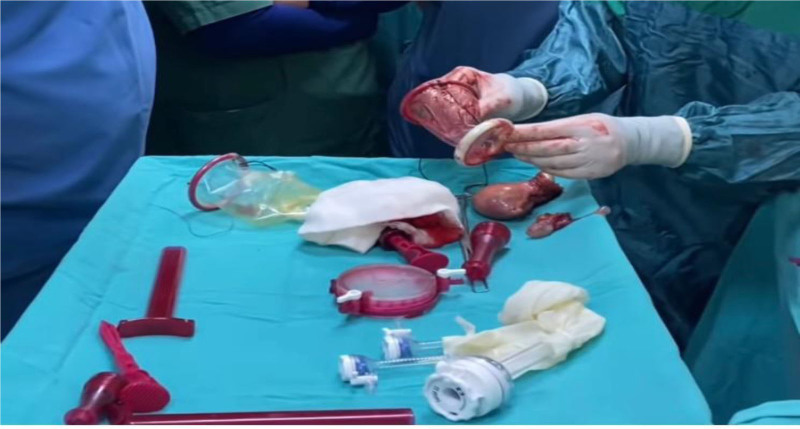
Gelpoint V-Path (applied medical) and manual Alexis retractor.

The cervix was grasped at the 12 and 6 lines with two single-toothed tenaculum. A paracervical circular incision was made with the help of cautery or a scalpel. The bladder in front and rectum in the back were detached and the visceral peritoneum was entered. Bilateral sacrouterine and cardinal ligaments were grasped and cut and tied, or burned and cut with the help of bipolar energy. After this stage, the Alexis retractor was deployed. In vNOTES hysterectomies performed in our center, we mostly use a manually prepared Alexis retractor. Gelpoint V-Path (Applied Medical) was used in 15 of 63 cases (Fig. 1).

The preparation of the Alexis retractor that we prepare manually is as follows: The aspirator hose is cut to wrap the surgeon’s 3 fingers contiguously in 2 turns, then sutured and fixed. A sterile surgical glove is cut 1 cm from the lateral parts of the 1st, 3rd, and 5th fingers and a gap is created. A 10 mm trocar is placed on the 1st and 5th finger and a 5 mm trocar is placed on the middle finger. These trocars are fixed (knotted) with pieces cut from the other glove pair. The wrist part of the trocar glove is prepared and passed through the inner part of the aspirator hose that is fixed and folded.

As for Gelpoint V-Path (Applied Medical), the 3 ports of the retractor in the gel part of the retractor are adjusted according to the surgeon.

After sacrouterine and cardinal ligaments are held and cut, the Alexis ring to be used is placed in the peritoneum. Four liters of CO_2_ is used to achieve pneumoperitoneum. The hysterectomy procedure is continued with laparoscopic bipolar energy. The resected uterus and the tuba and ovaries, if salpingo-oophorectomy was performed, are placed in the Alexis port and the Alexis port is removed from the peritoneum. The vaginal cuff is sutured individually or continuously with a no-1 absorbable synthetic monofilament suture.

### 2.2. Statistical analysis

The variables included in the descriptive statistics of the study were mean, standard deviation, and median. Compliance with normal distribution was checked with the Kolmogorov Simirnov normality test. Comparisons between groups were made using the One Way ANOVA for normally distributed variables, and the related subgroup comparisons were made with the Kurskal–Wallis test. In variables not suitable for normal distribution, Kruskal Wallis was employed, and comparisons between related subgroups were made with the Mann–Whitney *U* test. Comparisons between independent variables were made with an Independent samples *t* test. In all analyzes, *P* < .05 (two-way) was accepted as statistically significant. Spearman’s rank correlation coefficient was used for the correlation between variables. All data analyses were performed using SPSS version 21 (Statistics computing program, IBM Corporation).

## 3. Results

### 3.1. Demographic characteristics of the patients

The data regarding age, BMI, parity, history of cesarean section, history of pelvic surgery, and indications for hysterectomy are summarized in Table 1. When the three surgical techniques were compared, a significant difference was observed in terms of age (*P*: .01 < .05). When the three groups were evaluated separately, no significant difference was observed between vNOTES hysterectomy and TLH (*P*: 1.00 > .05), a significant difference was observed between vNOTES hysterectomy and VH (*P*: .01 < .05), as well as, between VH and TLH group (*P*: .01 < .05). When the BMI values of the three surgical techniques were compared, a significant difference was observed between the three surgical techniques (*P*: .008 < .05). When a pairwise comparison was made, a significant difference was observed between vNOTES hysterectomy and TLH (*P*: .005 < .05). There was no significant difference between vNOTES hysterectomy and VH (*P*: .459 > .05), as well as, between VH and TLH (*P*: .272 > .05). According to the results of our study, there was no relationship between parity and surgical techniques (*P*: 1139 > .05). There was a relationship between the history of cesarean section and surgical techniques (*P*: .01 < .05). There was no significant relationship between the history of pelvic surgery and surgical techniques (*P*: .662 > .05). Among the 186 patients who participated in our study, 31 (16.7%) patients were operated for myoma uteri, 71 (38.2%) for abnormal uterine bleeding, 18 (9.2%) for ovarian cysts, 57 (30.6%) for pelvic organ prolapse and 9 (4.8%) for cervical pathology. Abnormal uterine bleeding was the most common indication for the vNOTES hysterectomy and TLH group, while the most common indication for the VH group was pelvic organ prolapse with 47 patients (75.8%).

**Table 1:** Comparison of surgical techniques by demographic characteristics

Demographic characteristics	vNOTES hysterectomy group (n: 62)	Vaginal hysterectomy group (n: 62)	Laparoscopic hysterectomy group (n: 62)	*P* value between all groups	*P* value between vNOTES hysterectomy and TLH	*P* value between vNOTES hysterectomy-VH groups	*P* value between VH-TLH groups
Age (year)	51 ± 9	59 ± 10	50 ± 5	.01*	1.00	.01*	.01*
BMI (kg/m^2^)	29.8 ± 4.3	28.6 ± 4.3	27.9 ± 4.3	.008*	.005*	.459	.272
Parity	3.03 ± 1.4	3.19 ± 1.6	2.69 ± 1.3	1.139			
Those with a history of cesarean section	10 (16.1%)	4 (6.5%)	24 (38.7%)	.01*			
History of pelvic surgery	10 (16.1%)	7 (11.3%)	11 (17.7%)	.662			
Indication for hysterectomy				.01*			
Abnormal uterine bleeding*	29 (46.8%)	9 (14.5%)	33 (53.2%)				
Myoma uteri*	14 (22.6%)	3 (4.8%)	14 (22.6%)				
Overian cyst*	6 (9.7%)	1 (1.6%)	11 (17.7%)				
Pelvic organ prolapse*	10 (16.1%)	47 (75.8%)	0 (0.0%)				
Cervical pathology*	3 (4.8%)	2 (3.2%%)	4 (6.5%)				

TLH = total laparoscopic hysterectomy, VH = vaginal hysterectomy.

* *P* < .05.

### 3.2. Surgical results

Preoperative and postoperative Hb differences between the three groups were significant (*P*: .011 < .05). The vNOTES hysterectomy group had the highest Hb decrease (1.2 ± 0.9). Among the hysterectomy cases included in our study, the group with the lowest mean operation time was VH with 85 ± 29 minutes. The group with the highest operation time was the vNOTES hysterectomy group with 108 ± 41 minutes. There was no significant difference between vNOTES hysterectomy and TLH group (*P*: .57 > .05) and between VH and TLH group (*P*: .197 > .05), while there was a significant difference between vNOTES hysterectomy and VH group in terms of operation time (*P*: .005 < .05). The group with the lowest discharge time was the vNOTES hysterectomy group with 2.2 ± 0.8 days. The TLH group had the longest postoperative hospital stay with 2.5 ± 0.8 days. When the three groups were compared in terms of discharge times, a significant difference was observed between them (*P*: .018 < .05). When the three surgical techniques were compared in terms of uterine weight, no significant difference was observed (*P*: .426 > .05). When the adnexal surgery rates of the three surgical techniques were evaluated, the highest rate of salpingectomy and oophorectomy was found in the TLH group, and the rate of salpingectomy and oophorectomy of vNOTES hysterectomy was higher than VH. In the vNOTES hysterectomy group, bladder perforation was observed in 1 patient and primary bladder repair was performed by laparoscopy. No intraoperative complications were observed in the TLH group. In 3 VH operations, intraoperative complications were observed with bladder perforation in 2 patients and intraoperative bleeding in 1 patient. There was no significant difference in intraoperative complications between surgical techniques (*P*: .330 > .05). In our study, no postoperative complications were observed for all three surgical techniques. Three patients in the vNOTES hysterectomy group required conversion to laparoscopy or laparotomy. Bladder perforation was observed in one patient and a laparoscopy was performed. In one patient, laparoscopy was needed to control bleeding. A laparotomy was performed due to intraabdominal adhesions. There was no need for conversion to laparoscopy or laparotomy in the patient group who underwent TLH. In the VH group, laparoscopy was performed on 1 patient due to intraoperative bleeding. When the surgical techniques were compared, no significant difference was observed in terms of the need for additional surgery (*P* = .167 > .05). Sacrospinous colpopexy, cystocele repair, rectocele repair, and transobturator tape operations were also performed in some of the patients participating in our study according to their indications. Of 186 patients, 66 (35.5%) underwent additional surgical procedures and 120 (64.5%) did not undergo additional surgical procedures. When the three surgical techniques were evaluated separately; 16 (25.8%) patients in the vNOTES, 3 (4.8%) patients in the TLH group, and 47 (75.8%) patients in the VH group underwent additional surgical procedures. When the three surgical techniques were evaluated comparatively, a significant difference was observed in terms of additional surgical procedures (*P*: .01 < .05). We asked the patients who participated in our study to rate their pain intensity from 1 to 10 at the 6th postoperative hour and we used a numerical rating scale (NRS) for pain assessment. The average pain score of the patients who underwent a vNOTES hysterectomy operation was 1.53 (0–9), which is the lowest pain score. The average pain score for VH was 2.19 (0–6) and for TLH was 4.51 (0–9). When these three surgical techniques were compared, it was observed that there was a significant difference between them in terms of postoperative pain score (p:0.01 < 0.05). The overall surgical outcome table is summarized in Table 2.

**Table 2:** Comparison of surgical techniques according to surgical results

Surgical results	vNOTES hysterectomy group (n: 62)	Vaginal hysterectomy group (n: 62)	Laparoscopic hysterectomy group (n: 62)	*P* value between all groups	*P* value between vNOTES hysterectomy and TLH	*P* value between vNOTES hysterectomy-VH Groups	*P* value between VH-TLH groups
The difference between preoperative and postoperative Hb	1.2 ± 0.9	1.7 ± 0.8	1.6 ± 1.0	.011*	.133	.009*	.535
The duration of the operation (min)	108 ± 41	85 ± 29	100 ± 42	.007*	.57	.005*	.197
Duration of discharge (d)	2.2 ± 0.8	2.3 ± 0.8	2.5 ± 0.8	.018*	.022*	1.00	.096
Uterine weight (g)	77 ± 25	79 ± 27	89 ± 42	.426			
Cost (Turkish Lira)	5.519 ± 3.677	3.491 ± 1.846	11.125 ± 5.949	.01*	.01*	.003*	.01*
Oophorectomy	46 (74.2%)	34 (54.8%)	55 (88.7%)	.01*			
Salpingectomy	56 (90.3%)	30 (48.4%)	61 (98.4%)	.01*			
Postoperative complication	0 (0.0%)	0 (0.0%)	0 (0.0%)				
Intraoperative complication	1 (1.6%)	3 (4.8%)	0 (0.0%)	.330			
Additional surgical procedure	16 (24.2%)	47 (75.8%)	3 (4.8%)	.01*			
The need for conversion to laparoscopy or laparotomy	3 (4.8%)	1 (1.6%)	0 (0.0%)	.167			
Postoperative 6 hour pain score				.01*			
0	21 (33.9%)	13 (21%)	2 (3.2%)				
1	15 (24.2%)	7 (11.3%)	0 (0.0%)				
2	15 (24.2%)	23 (37.1%)	6 (9.7%)				
3	6 (9.7%)	13 (21%)	13 (21%)				
4	1 (1.6%)	1 (1.6%)	4 (6.5%)				
5	1 (1.6%)	3 (4.8%)	18 (29.0%)				
6	1 (1.6%)	2 (3.2%)	14 (22.6%)				
7	0 (0.0%)	0 (0.0%)	2 (3.2%)				
8	1 (1.6%)	0 (0.0%)	2 (3.2%)				
9	1 (1.6%)	0 (0.0%)	1 (1.6%)				
10	0 (0.0%)	0 (0.0%)	0 (0.0%)				

TLH = total laparoscopic hysterectomy, VH = vaginal hysterectomy.

*P < .05.

## 4. Discussion

Hysterectomy is a very common surgical treatment in gynecologic surgery.^[10]^ The most important factor in postoperative morbidity after a hysterectomy is the surgical technique. There are numerous studies evaluating surgical approaches and complications according to the type of operation to determine which procedure is best for the patient.^[11]^

In our study, we compared the minimally invasive hysterectomy techniques (TLH, vNOTES hysterectomy, VH) performed in our clinic and aimed to evaluate which minimally invasive hysterectomy procedure is best in terms of the appropriateness of preoperative evaluation for patient benefit.

Laparoscopic hysterectomy and vaginal hysterectomy have been frequently compared in the literature, but vNOTES hysterectomy is a newer technique and therefore less research has been devoted to it.

In many studies, vaginal hysterectomy has been found to be superior to abdominal and laparoscopic hysterectomy, and vaginal hysterectomy has been accepted as the gold standard hysterectomy technique.^[2,12]^ Studies have shown that vaginal hysterectomy is shorter than laparoscopic and abdominal hysterectomy and postoperative discharge time is shorter in vaginal hysterectomy. This also reduces the cost. Vaginal hysterectomy also avoids trocar access complications. It also provides the patient with scarless surgery.

Intra-abdominal adhesions, history of pelvic surgery, lack of descensus uteri, endometriosis, previous pelvic inflammatory diseases, and intra-abdominal adhesions are barriers to the use of vaginal hysterectomy.^[13]^ In addition, large uterine volume made retraction difficult in vaginal hysterectomy. Surgeons often resorted to laparoscopic hysterectomy or abdominal hysterectomy in patients with large uterine volumes. At the same time, access to the adnexa was not always possible in vaginal hysterectomy.

In vNOTES hysterectomy, although the large uterine volume makes the procedure difficult, it has also been used in patients with large uterine volumes to provide a clearer view for the surgeon. At the same time, it is observed to facilitate hysterectomy in patients without uterine descensus compared to vaginal hysterectomy and is thought to make it more possible to reach hard-to-reach adnexa with a clearer view.^[14]^

The first vNOTES hysterectomy case was performed by Su et al.^[3]^ The included hysterectomies were fulfilled without the need for any additional surgery. Hysterectomies with benign indications were included in the study. Mean uterine weight was 538.8 ± 102.9 g, mean operative time was 122.7 ± 17.6 minutes, and mean blood loss was 379.4 ± 95.4 ml. Postoperative discharge time was calculated as 2.8 ± 0.2 days. This study is important because it is the first study indicating that vNOTES hysterectomy can be used as an alternative to laparoscopic and vaginal hysterectomy in hysterectomies with benign indications.

In a study published by Nulens et al, 114 cases of vNOTES with uterine weights of 280 g or more operated at the Belgian Training Hospital with benign indications were retrospectively evaluated.^[4]^ In this study, uterine weights ranged between 281 g and 3361 g. The mean surgical time was 63 ± 34 min and it was found that the mean surgical time was directly proportional to the uterine weight. In addition, no complications were observed in this case series. The study showed that vNOTES hysterectomy can be a safe surgical alternative to laparoscopy or laparotomy in cases with large uterus even if the patient has a history of cesarean section, nulliparity, or obesity.^[4]^ In our study, there was no significant difference between uterine weight and operation time (*P*: .68 > .05). In addition, when uterine weights of the three surgical techniques were compared in our study, no significant difference was observed (*P*: .426 > .05). This supports that vNOTES hysterectomy can also be used safely in patients with large uterine weights.

Kaya et al compared vNOTES hysterectomy and TLH in a series of 99 cases.^[5]^ Operative times, intraoperative and postoperative complications, visual analog scale (VAS) scores at 6 and 24 hours postoperatively and hospitalization times of the two minimally invasive methods were evaluated. The mean operation time of the vNOTES hysterectomy group (79.56 ± 32.54 min) was shorter than that of the TLH group (120.67 ± 38.35 min) and was significantly different. In addition, the postoperative hospital stay of the vNOTES hysterectomy group was significantly shorter than that of the TLH group. These findings have shown that vNOTES hysterectomy may be a viable alternative to TLH.^[5]^ In our study, the mean operation time for vNOTES hysterectomy was 108 ± 41 min, while the mean operation time for TLH was 100 ± 42 min; no significant difference was observed between vNOTES hysterectomy and TLH in terms of operation time (*P*: .57 > .05). We attribute the reason why the mean operation time of vNOTES hysterectomy was higher in our center to the fact that it is a newly performed operation in our center. When the two operations were compared in terms of discharge time in our study, we observed that the patients who underwent vNOTES hysterectomy were discharged significantly shorter than the TLH group (*P*: .022 < .05), supporting the study of Kaya et al.

Merlier et al compared vNOTES hysterectomy and vaginal hysterectomy with a series of 50 cases.^[6]^ In the results of the study, vNOTES hysterectomy was ascertained superior to vaginal hysterectomy in terms of ease of access to the adnexa. No significant difference was found between the two operations in terms of uterine weights and outpatient surgery. In our study, vNOTES hysterectomy was superior to VH in terms of performing oophorectomy and salpingectomy operations. There was no significant difference between the two surgical techniques in terms of postoperative discharge time in our study (*P*: 1.0 > .05).

In our study, we concluded that we had preferred vNOTES hysterectomy more than vaginal hysterectomy in patients with a history of previous cesarean section and/or pelvic surgery. This is because our field of view is wider in vNOTES hysterectomy compared to VH, so intraabdominal adhesions can be seen more clearly. TLH was the most preferred surgical technique in patients with possible intraabdominal adhesions. Our study did not include patients who underwent surgery due to malignancy or endometriosis.

Intraoperative complications were observed in 4 patients in our study. One bladder perforation was observed in the vNOTES hysterectomy operation, while two bladder perforation and one bleeding was observed in the VH group. There were no complications in the TLH group.

When we compared the surgical techniques in our study in terms of BMI, patients who underwent vNOTES hysterectomy were found to be significantly higher than patients who underwent TLH (*P*: .005 < .05). There was no significant difference between vNOTES and VH in terms of the BMI values of the patients (*P*: .459 > .05). Laparoscopic hysterectomy in obese patients is technically more difficult than vaginal hysterectomy. Blockage of the image by omentum fatty tissue and difficulty in accessing the deep pelvis with laparoscopic instruments are some of the reasons that make TLH difficult in obese patients. Although exclusion becomes difficult in the vaginal route, we think that the vaginal route is superior to the laparoscopic route in patients with high BMI.

When the average cost of the operations included in our study is calculated, we realize that VH is significantly less costly than vNOTES hysterectomy and TLH (*P*: .01 < .05). The costliest operation was TLH. vNOTES hysterectomy was significantly less costly than TLH (*P*: .01 < .05).

## 5. Conclusion

In our literature review, vNOTES hysterectomy was retrospectively compared with a vaginal hysterectomy and laparoscopic hysterectomy separately, but no study comparing these three surgical techniques together was found. Our study will be helpful in clinical practice by comparing these three surgical techniques, which have an important place in minimally invasive hysterectomy techniques used in our clinics, in a single center study.

In conclusion, vNOTES hysterectomy is a safe surgical method to be preferred because it has less Hb decline, shorter postoperative discharge time, and lower pain score compared to TLH and VH. In addition, in our study, vNOTES hysterectomy was superior to VH in accessing the adnexa. In patients with previous intra-abdominal surgery, vNOTES hysterectomy can be used more safely than VH. Although retraction is difficult in vaginal hysterectomy in patients with a large uterus, vNOTES hysterectomy provides a wider view than VH and can be used safely in patients with a large uterus. In addition, the cost of vNOTES hysterectomy is lower than TLH and it is a more aesthetic operation without incisions using the vaginal route. Aesthetic results can be achieved for patients by using the vaginal route.With all these results, we believe that vNOTES hysterectomy is a safe alternative to TLH and VH.

## Author contributions

**Conceptualization:** Fatmanur Mollahüseyinoğlu Küllaç, Ayşe Zehra Özdemir.

**Data curation:** Ayşe Zehra Özdemir.

**Methodology:** Fatmanur Mollahüseyinoğlu Küllaç, Ayşe Zehra Özdemir, Çağlanur Yildiz.

**Project administration:** Ayşe Zehra Özdemir.

**Resources:** Fatmanur Mollahüseyinoğlu Küllaç, Ayşe Zehra Özdemir.

**Validation:** Ayşe Zehra Özdemir.

**Visualization:** Fatmanur Mollahüseyinoğlu Küllaç, Çağlanur Yildiz.

**Writing – original draft:** Fatmanur Mollahüseyinoğlu Küllaç.

**Writing – review & editing:** Fatmanur Mollahüseyinoğlu Küllaç, Ayşe Zehra Özdemir, Çağlanur Yildiz.
